# FAIRer DNA sequence data with q2-ena-uploader: a QIIME 2 plugin for data deposition in the European Nucleotide Archive

**DOI:** 10.1128/mra.01155-25

**Published:** 2026-01-12

**Authors:** Zuzana Sebechlebska, Michal Ziemski, Nicholas A. Bokulich

**Affiliations:** 1 Department of Health Sciences and Technology, ETH Zurich 27219, Zurich, Switzerland; University of Michigan, Ann Arbor, Michigan, USA

**Keywords:** FAIR data, DNA sequencing, data submission, QIIME 2

## Abstract

Technical hurdles are a significant barrier for deposition of next-generation sequence data in public repositories. We present q2-ena-uploader, a software package for automated validation and upload of sequence data. It is BSD-3-licensed and available at https://github.com/bokulich-lab/q2-ena-uploader.

## ANNOUNCEMENT

The pace of NGS data generation continues to accelerate, yet a substantial share of that data never makes it into public repositories like the European Nucleotide Archive (ENA) or Sequence Read Archive (SRA) (1). While many scientific journals now encourage or even require raw sequence deposition, practical hurdles often hinder researchers (2). The submission process can be time-consuming, technically complex, and difficult to navigate, frequently requiring familiarity with specific metadata standards and command-line tools/interfaces. Consequently, a significant fraction of valuable data remains inaccessible to the broader research community (3), actively hindering efforts to make scientific data Findable, Accessible, Interoperable, and Reusable (FAIR) (4).

We developed q2-ena-uploader to address some of these challenges. This new plugin was developed as a plugin for the popular microbial bioinformatics framework QIIME 2 (5) to streamline the entire process of uploading raw NGS data to the ENA repository. QIIME 2’s modular, plugin-based architecture and diverse user interfaces make it an ideal framework to lower technical barriers for researchers already familiar with its ecosystem and to encourage broader data sharing. We chose ENA due to its support for programmatic data upload and comprehensive documentation, both crucial for developing a seamless tool. As ENA and SRA exchange data via the International Nucleotide Sequence Database Collaboration (INSDC), ENA provides a convenient entry point for data deposition in the INSDC.

**Fig 1:**
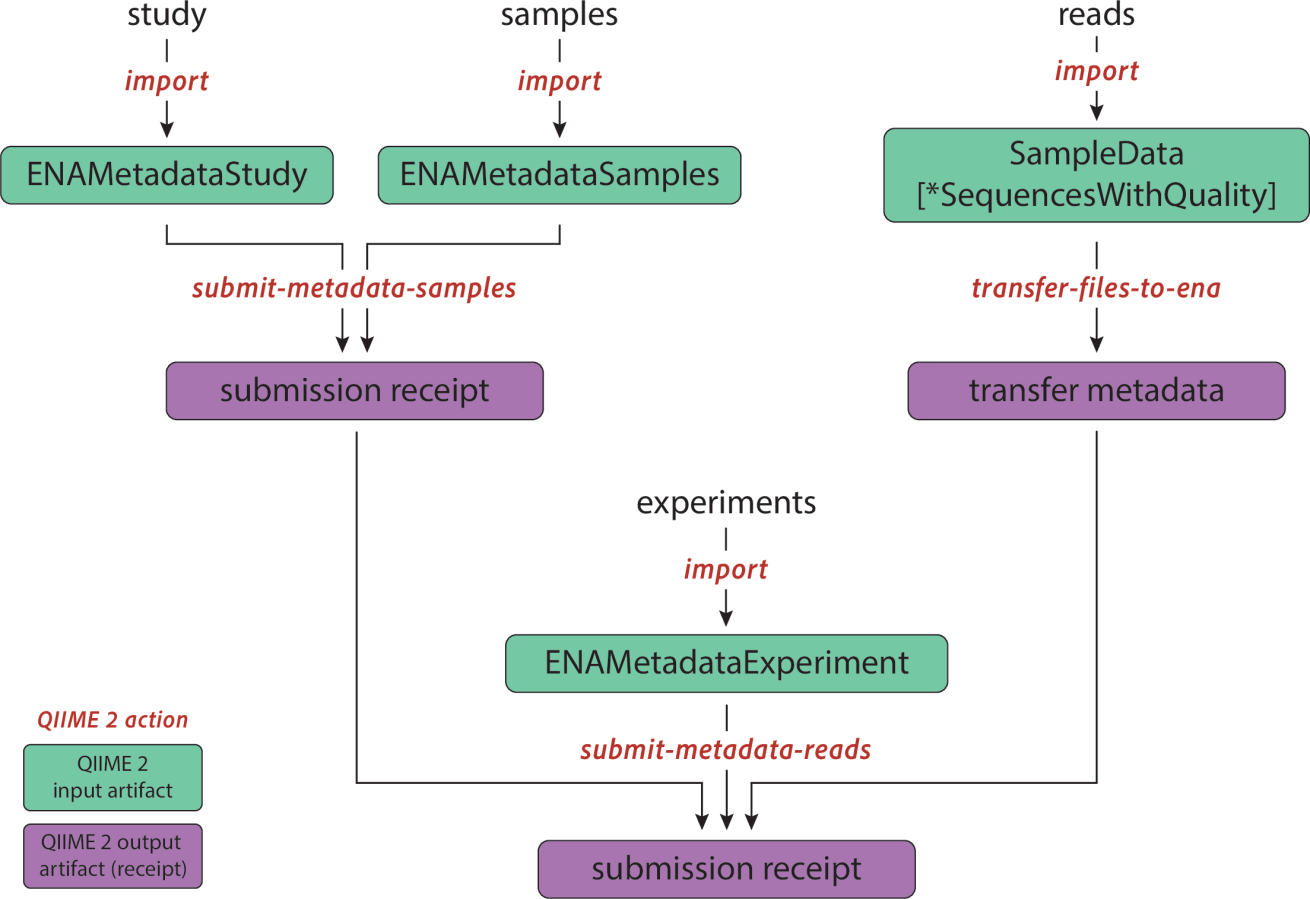
Overview o*f actio*ns available in the q2-ena-uploader QIIME 2 plugin. Studies and samples can be submitted using th*e submit-metadata-samples* action, while the experiment metadata can be submitted using th*e submit-metadata-reads* action. The raw data files themselves can be uploaded using th*e transfer-files-to-ena* action. Each action provides a unique receipt as an output, which can be investigated for potential errors or to access the assigned accession numbers.

The q2-ena-uploader plugin offers individual actions for uploading study, sample, and run metadata, as well as the raw data files (Fig. 1). Furthermore, it provides a single, integrated pipeline that chains these steps together, enabling users to submit their entire experiment with a single command, provided all required metadata is present. A key strength of this approach is the robust data validation provided by the QIIME 2 framework—the plugin validates all metadata for studies, samples, and experiments to ensure that the necessary standard columns required for a successful ENA submission are present and correctly formatted. Sample IDs are cross-checked across different submission steps, guaranteeing data integrity throughout the process. Any detected issues, whether related to raw data integrity or missing metadata, are flagged as errors, allowing users to address them before submission reaches ENA servers. Finally, all ENA submission receipts are stored as unique QIIME 2 artifacts, enabling users to easily retrieve submission information, including assigned accession numbers, at any time.

The development of q2-ena-uploader significantly reduces the technical burden associated with public data deposition, enabling more researchers to comply with data sharing mandates and contribute to open science initiatives. For existing QIIME 2 users, this integration is particularly powerful: their data is already managed as QIIME 2 artifacts, so submitting to ENA becomes just another logical step in their workflow, leveraging familiar tools and interfaces. For researchers who are not currently QIIME 2 users, q2-ena-uploader can still simplify the often-complex submission process by providing streamlined workflows accessible via the different user interfaces of QIIME 2. Overall, our plugin enables researchers to easily share their NGS data with the scientific community, promoting FAIR research data practices. We believe q2-ena-uploader will play a crucial role in accelerating scientific discovery by ensuring availability of high-quality data and reducing temporal and technical hurdles to sharing research data.

## Data Availability

The q2-ena-uploader plugin is freely available (BSD-3 license) at https://github.com/bokulich-lab/q2-ena-uploader. The q2-ena-uploader repository is publicly archived on Zenodo under the DOI https://doi.org/10.5281/zenodo.17642489. The plugin is implemented in Python and supports all operating systems compatible with QIIME 2: Linux and macOS natively, and Windows via WSL2 or Docker environments.

## EDITOR'S NOTE

**Ed. Note**: This article was handled by Pat Schloss, who acted as a Guest Editor in consultation with the Editor in Chief, Irene Newton. Dr. Schloss's role as the Guest Editor of this article is outside of his duties as the chair of the Journals Committee.
